# Supplementary material for: Notch and retinoic acid signals regulate macrophage formation from endocardium downstream of Nkx2-5
Source: Nat Commun. 2023 Sep 5;14:5398. doi: 10.1038/s41467-023-41039-6 (PMC10480477; doi:10.1038/s41467-023-41039-6)
Supplement: Supplementary file 1 — Supplementary Information [file 41467_2023_41039_MOESM1_ESM.pdf]

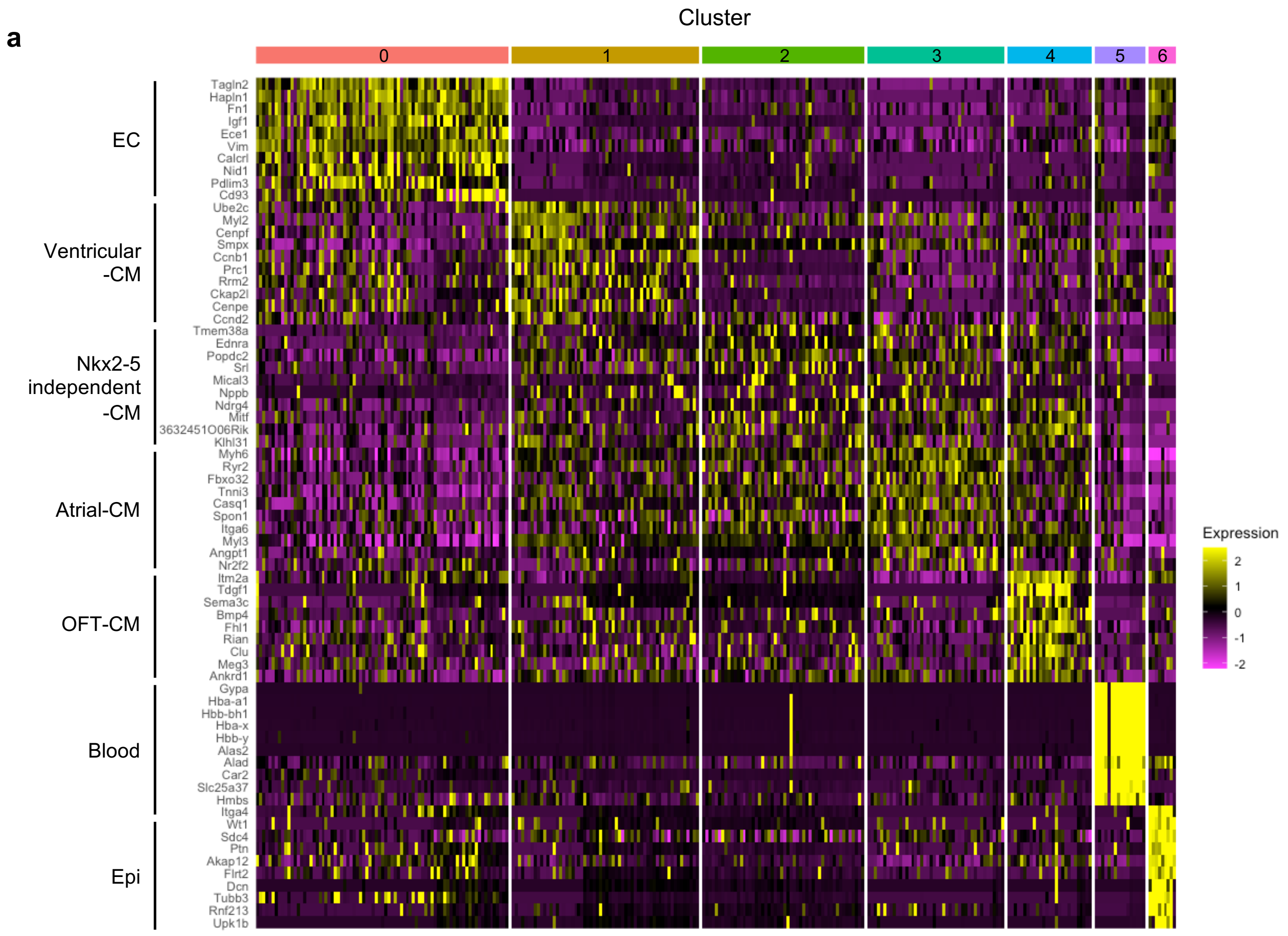

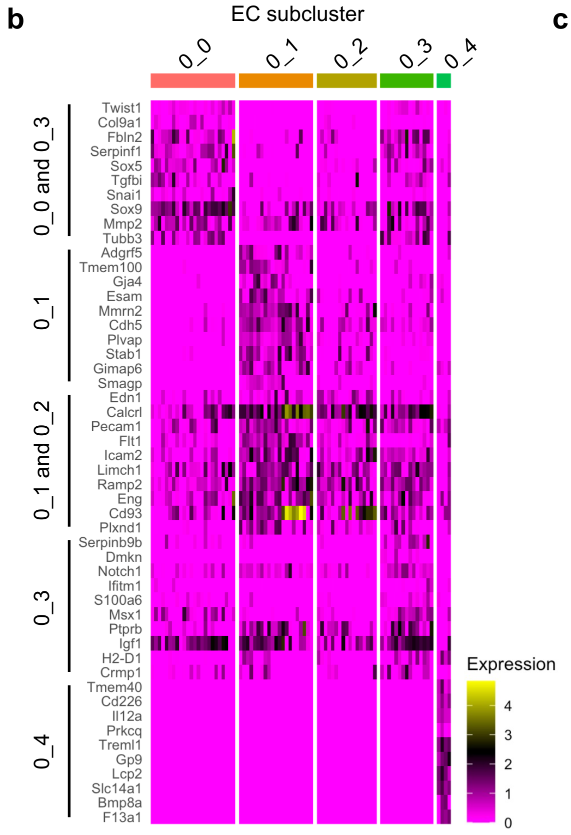

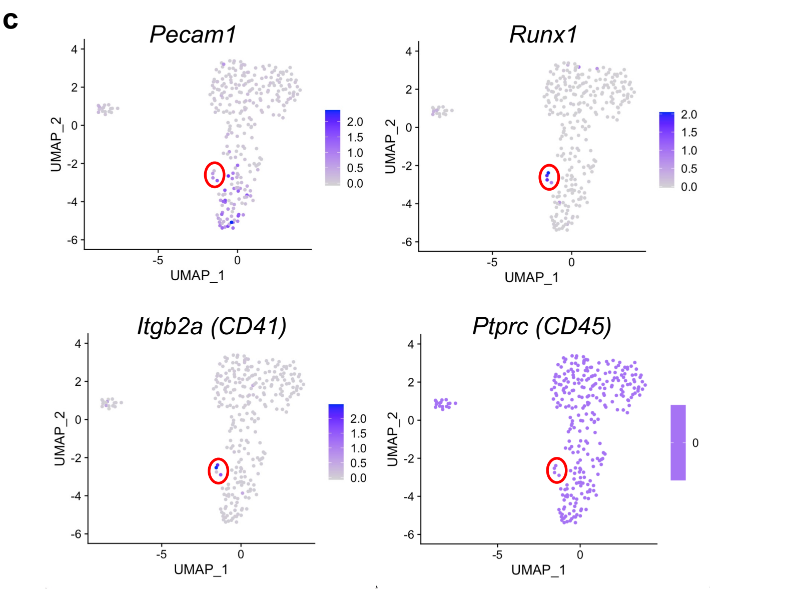

Supplementary Figure 1. Heatmap of unsupervised clustering analysis featuring the top 10 discriminative genes per cluster. **(a)** Marker genes (defined by log2 fold change) of cluster 0 to 6. EC: endocardial/endocardial cell, CM: cardiomyocyte, OFT-CM: outflow tract cardiomyocyte, Blood: blood cell (leukocyte), Epi: pro-epicardial cell **(b)** Marker genes (defined by adjusted p-value, based on bonferroni correction using all genes in the dataset) of endocardial/endothelial cell subcluster 0_0 to 0_4. **(c)** Feature plots of Pecam1, Runx1, CD41, and CD45. The indicated subcluster 0_4 (circled with red) expresses Pecam1, Runx1, and CD41, but not CD45.

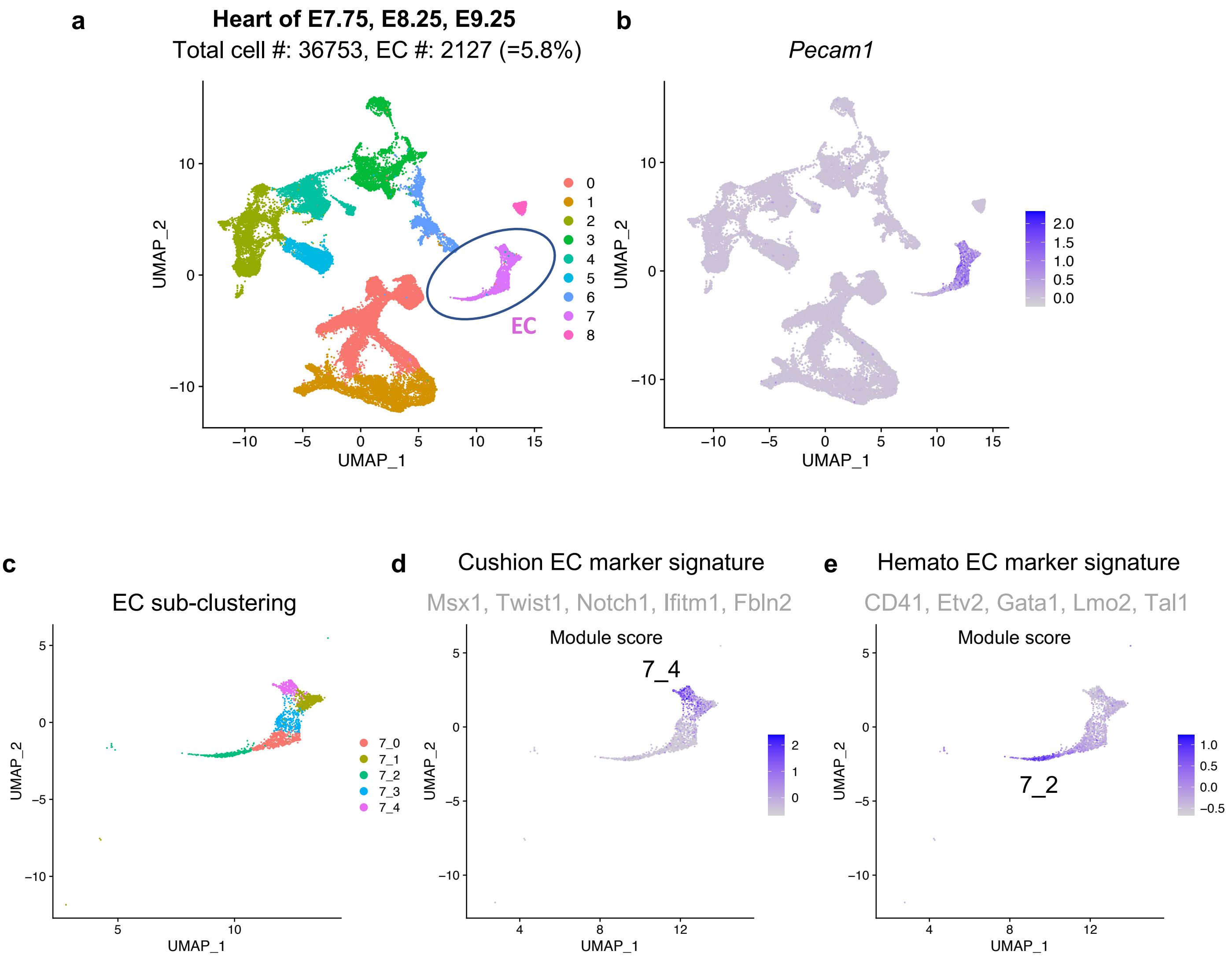

Supplementary Figure 2. Cushion endocardial cells and hematopoietic progenitors are present in the hearts of E7.75 to E9.25 mice. (**a**) UMAP representation of single-cell gene expression from wild-type mouse hearts of three gestational stages (GSE126128). **(b)** UMAP feature plot showing a high expression of *Pecam1* in cluster 7. (**c**) UMAP representation of single-cell gene expression showing that the endocardial cluster can be subclustered into five (7_0 to 7_4). (**d**) (**e**) Module score projected on the UMAP demonstrating that cluster 7_4 was enriched for cushion endocardium marker genes (*Msx1, Twist1, Notch1, Ifitm1,* and *Fbln2*) (d) and cluster 7_2 was enriched for hematopoietic progenitor marker genes (*CD41(Itga2b), Etv2, Gata1, Lmo2,* and *Tal1*) (e).

a

Genes included in “Notch signaling pathway” (GO: 007219)	
Notch1	*Pln*
Rbpj	*Postn*
Il6st	*Psen1*
Jag1	*Psen2*
Ncstn	*Traf7*
Pgam2	*Tspan14*

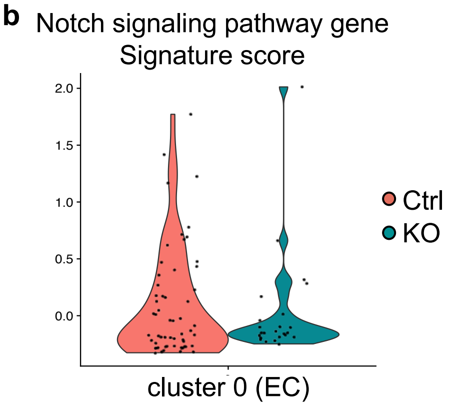

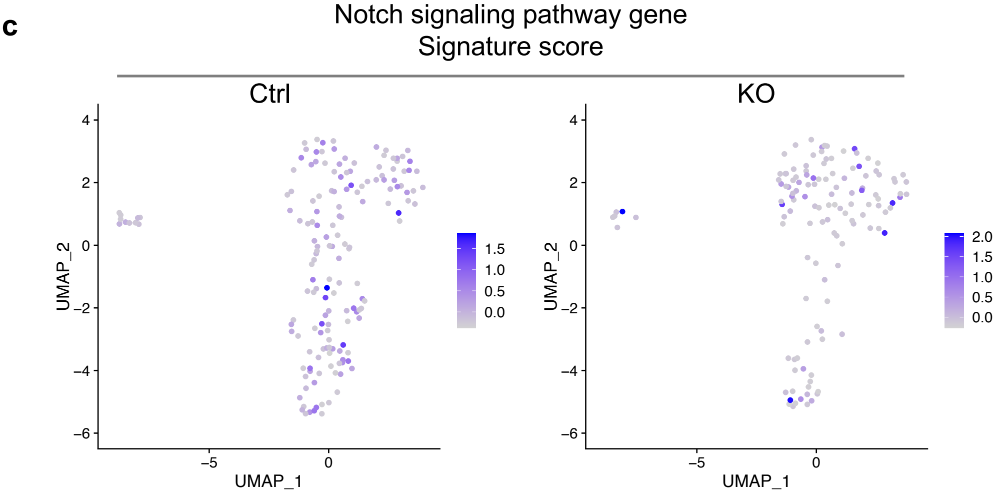

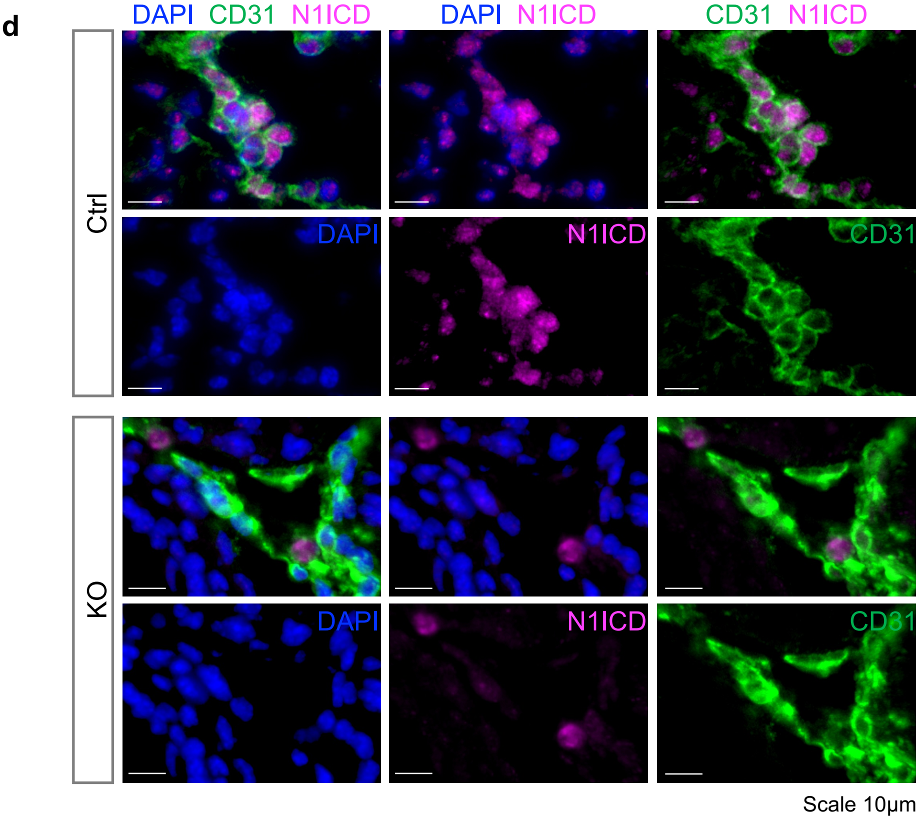

Supplementary Figure 3. Notch signaling pathway related genes are downregulated in KO endocardium. (**a**) List of genes in the GO term 007219 "Notch signaling pathway" that are expressed in the scRNA-seq data. (**b**) Violin plot shows signature scores of Notch signaling pathway related genes. The plot is generated using AddModuleScore function in R. (**c**) UMAP feature plots of signature score of Notch signaling pathway related genes in Ctrl and KO. **(d)** N1ICD expression in endocardial cells of Ctrl and KO embryos. The images correspond to the cropped images shown in Figure 2C(c).

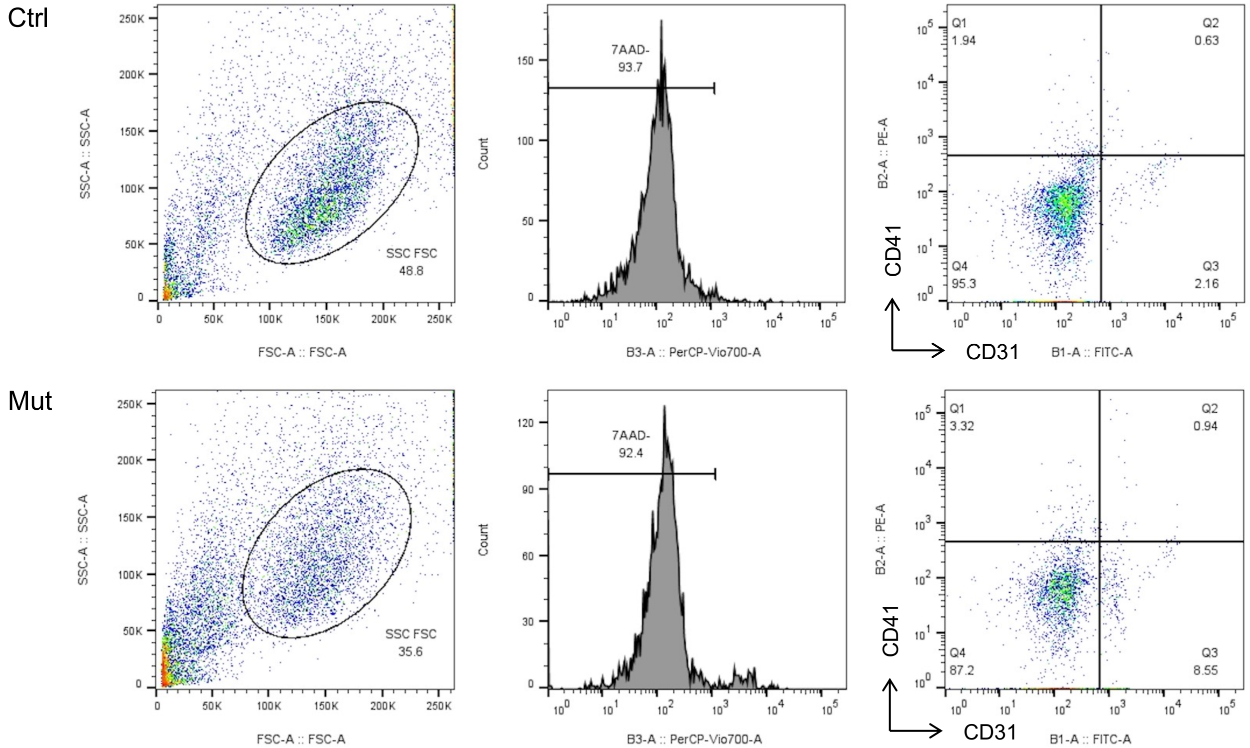

Supplementary Figure 4. Flow cytometry profile for the quantification of CD31$^+$ CD41$^+$ cells shown in Figure 2. Representative flow cytometry gating strategy of hematopoietic endocardial cells detected in the hearts of E10.0 Ctrl and *Nkx2-5*$^{cre/+}$*; R26*$^{NICD\text{-}GFP/+}$ (Mut) embryos.

a

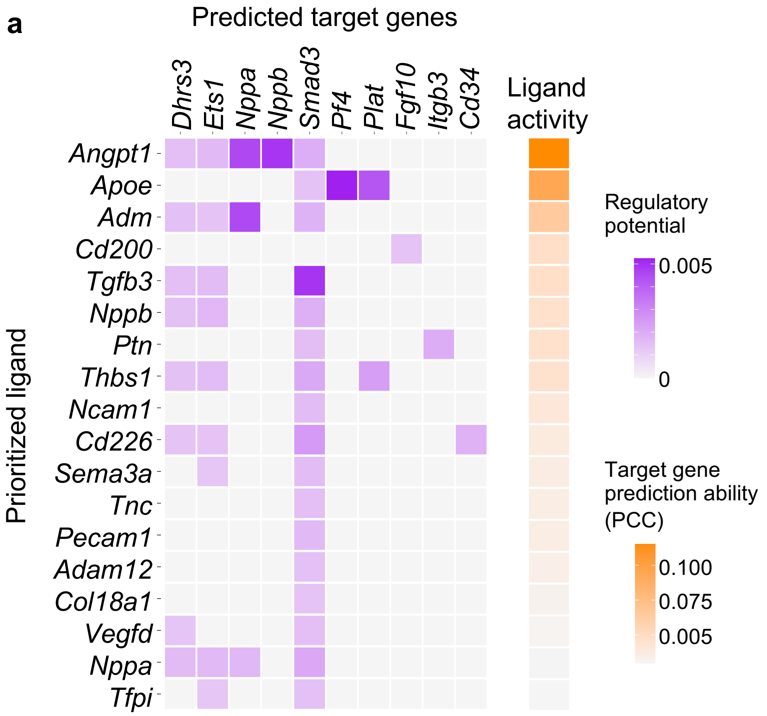

Supplementary Figure 5. Intercellular and intracellular gene regulatory networks that are significantly different between Ctrl and KO. (**a**) Outcome of NicheNet's ligand-target matrix resulted from the DEGs between Ctrl and KO in EC and CM. Result of Ligand activity prediction calculated by Pearson correlation coefficient (PCC) is shown on the right. (**b**) NicheNet result of prioritized ligands and their corresponding receptor interactions. Receptor genes were pulled from the DEGs between Ctrl and KO, and the interaction potentials were matched from weighted ligand-receptor network database provided by NicheNet.

b

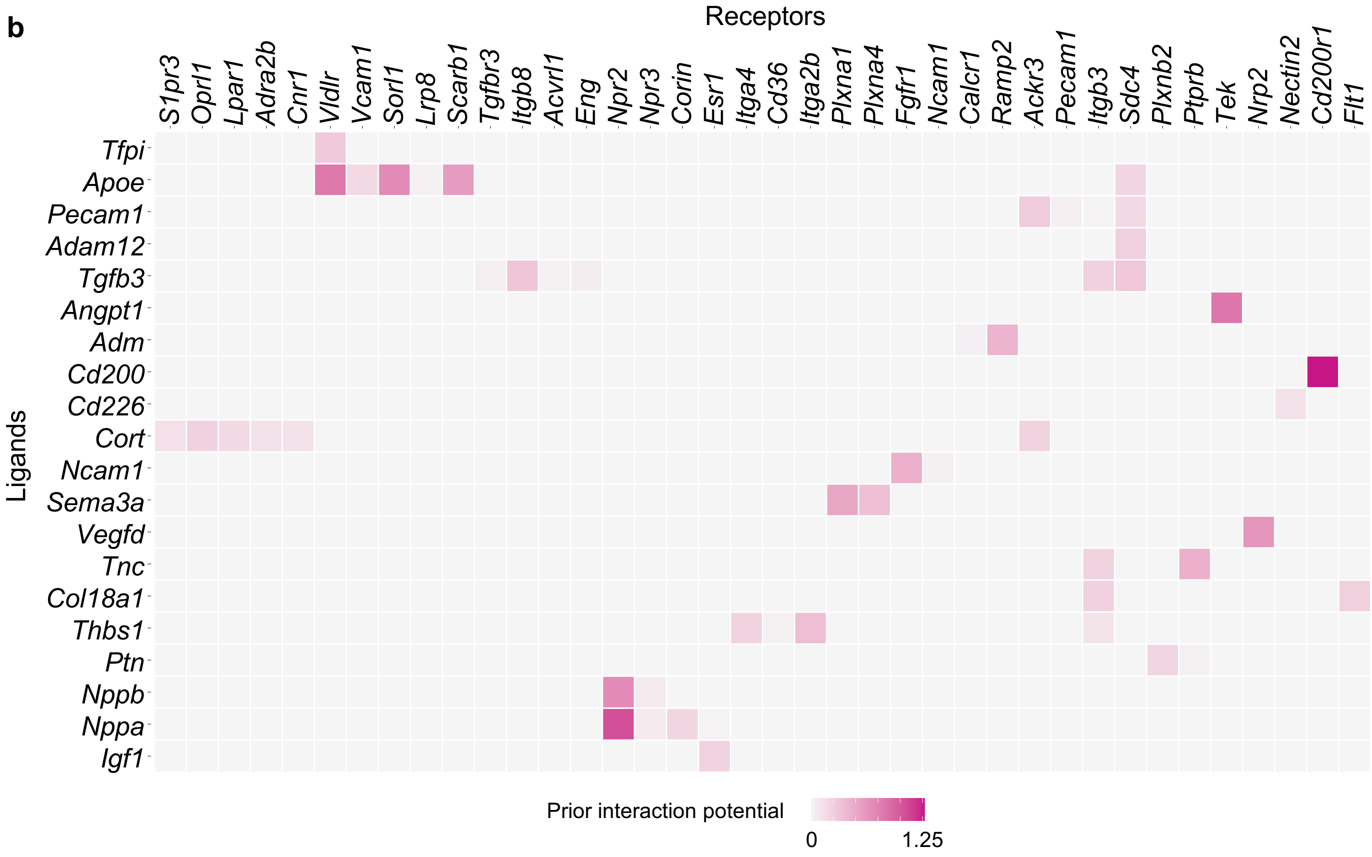

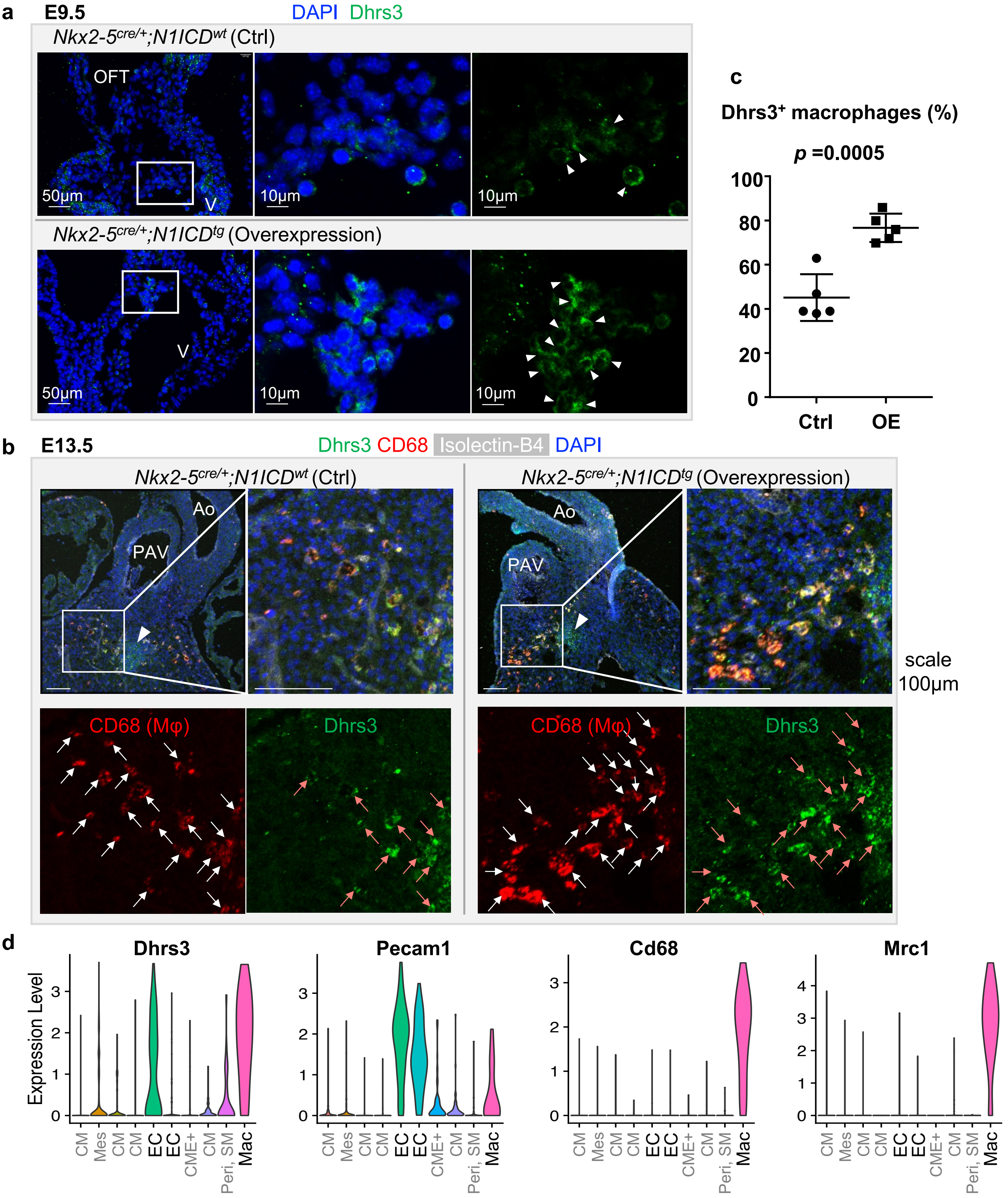

Supplementary Figure 6. Forced activation of Notch signaling increases the number of Dhrs3⁺ endocardial cells (E9.5) and macrophages (E13.5). (**a**) Representative picture of section immunofluorescent staining of Dhrs3 in E9.5 hearts of Ctrl and Overexpression embryos. Enlarged view of endocardial cells in ventricle region showed the increase of Dhrs3$^+$ cells (white arrow head) in Overexpression embryo. Three experiments were repeated with similar results. OFT: outflow tract, V: ventricle **(b)** Representative picture of section immunofluorescent staining of Dhrs3, CD68 and Isolectin-B4 in E13.5 hearts of Ctrl and Overexpression embryos. The identification of regions of the cardiac cushion equivalent in Ctrl and Overexpression was determined by confirming the presence of distinct apoptotic cell debris (white arrow head). **(c)** Quantified result of Dhrs3$^+$ macrophages in the cardiac cushion in Ctrl and Overexpression embryos at E13.5. Data are presented as mean values +/- SEM. Five embryos were analyzed in each group (n=5), *p*=0.0005 from unpaired, two-tailed t-test. Source data are provided as a Source Data file. **(d)** Violin plots showing Dhrs3 expresses in both endothelial cells and macrophages throughout embryonic and neonatal stages (E9.5, E11.5, E14.5, E18.5, P0, P3, P7, and P21). The scRNA-seq data was obtained from Ref 30. CM: cardiomyocyte, Mes: mesenchyme, EC: endothelial cell, CME+: cardiomyocytes expressing extracellular matrix proteins, Peri, SM: pericyte, smooth muscle cell, Mac: macrophage. These annotations were based on the original paper (Ref 30).

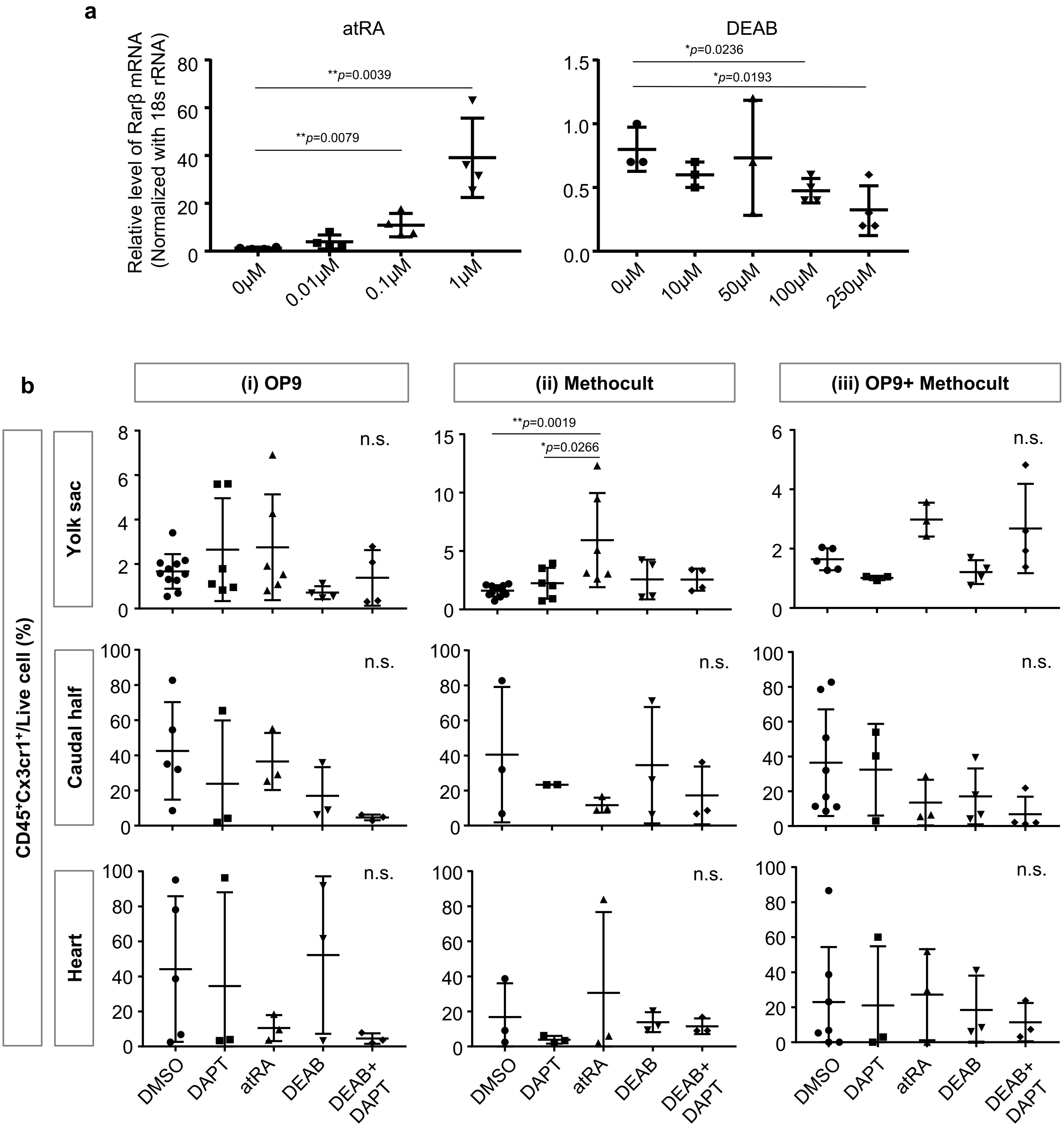

Supplementary Figure 7. Effects of Notch and RA signals in hematopoietic colony formation. (a) Retinoic acid receptor-β (RARβ) mRNA expression levels in response to different doses of atRA and DEAB. Data are presented as mean values +/- SEM. Each dot plot represent result from different embryo (n=3 or 4), *p* values were calculated from unpaired, two-tailed t-test. **(b)** Ratio of $CD45^{+}$ $Cx3cr1^{+}$ macrophages grown from E8.25 tissue explants analyzed by flow cytometry. Data are presented as mean values +/- SEM. Yolk sac: Three independent experiments with 2 replications in each condition were performed (n=3). Caudal half: Three independent experiments (5 embryos were pooled for each experiments) in each condition were performed (n=3). Heart: Three independent experiments (5 embryos were pooled for each experiments) in each condition were performed (n=3)., *p* values were calculated by One-way ANOVA with post-hoc Tukey's multiple comparisons test. Source data are provided as a Source Data file.

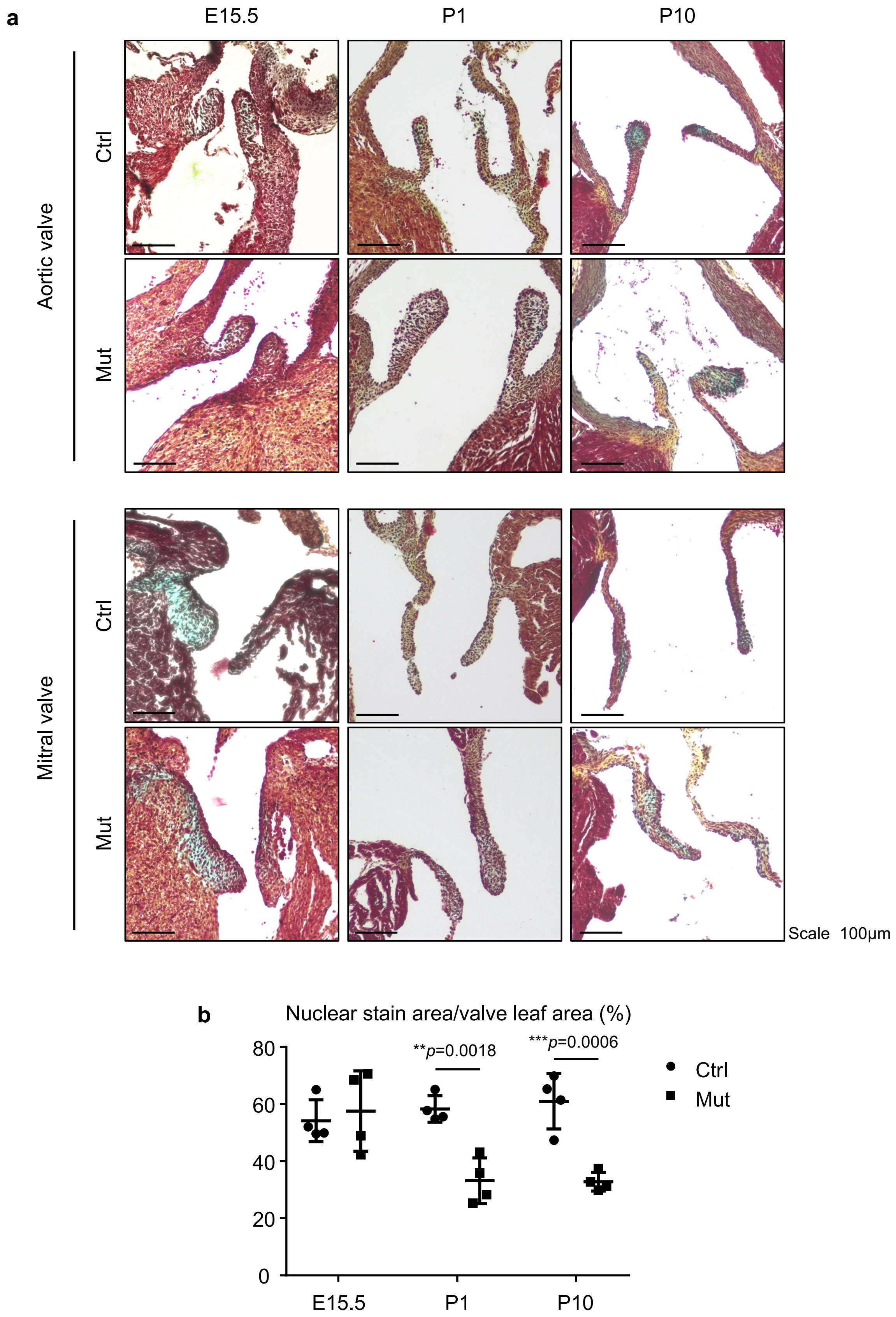

Supplementary Figure 8. Cardiac valve remodeling defects in ablation of Csf1r (macrophage surface marker) in Nkx2-5 lineage appear after birth. (a) Pentachrome staining of aortic and mitral valves of control (Ctrl) and mutant (Mut) mice at E15.5, P1, and P10; Mut valves were thicker than Ctrl valves at P1 and P10, but no obvious difference at E15.5. **(b)** Extracellular matrix accumulation was compared by calculating the percentage of nuclei to valve leaf area. In P1 and P10, the staining area of nuclei was significantly reduced in Mut, suggesting increased deposition of non-cellular regions in Mut. Data are presented as mean values +/- SEM. Representative sections from 4 animals were quantified (n=4), ***p*=0.0018, ****p*=0.0006. Sidak's multiple comparisons test were performed between Ctrl and Mut at respective stages. Source data are provided as a Source Data file.

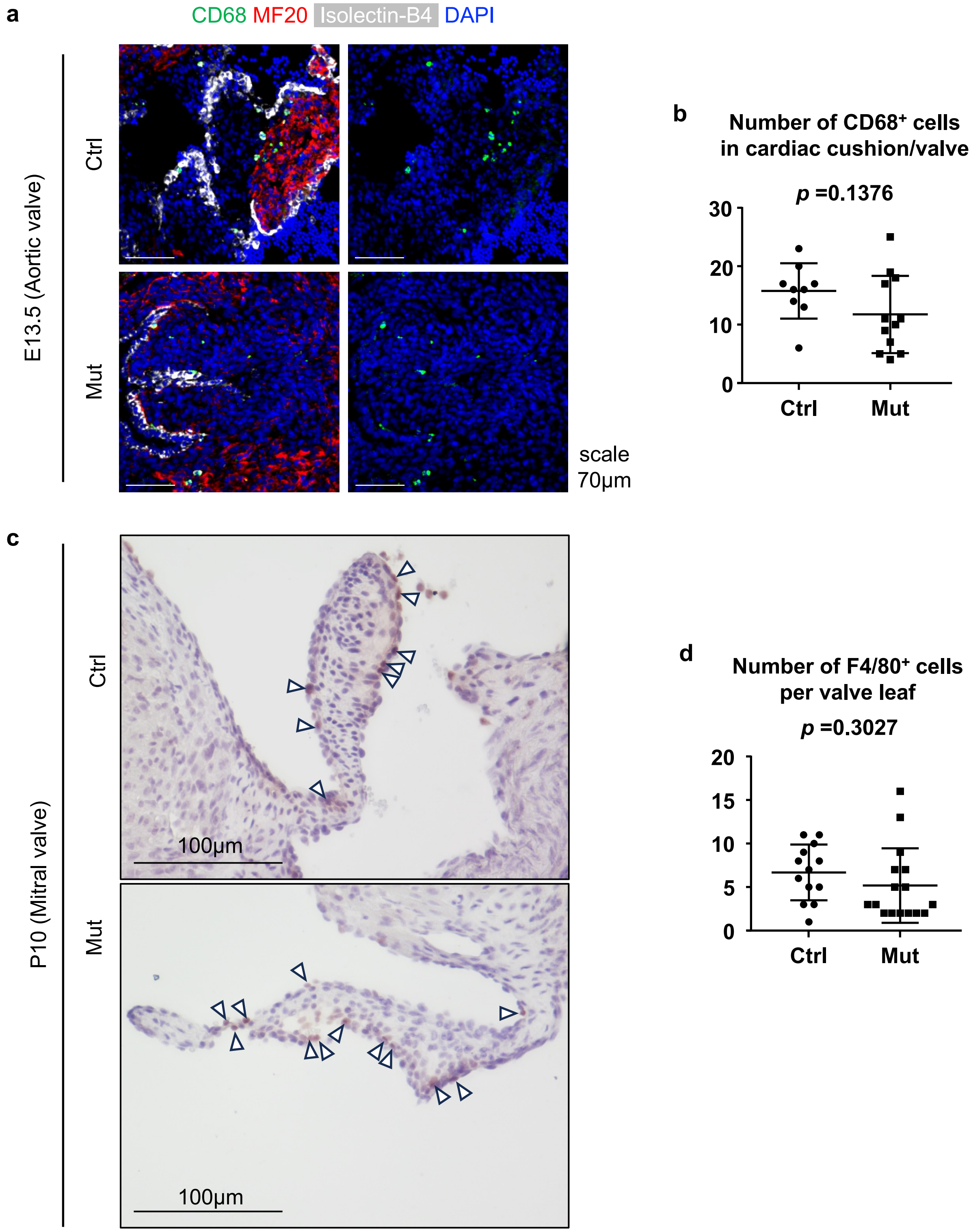

Supplementary Figure 9. Number of macrophages in the cardiac valves and cushion area. (a) Representative images of section immunofluorescence staining of aortic valves of control (Ctrl) and mutant (Mut) mice at E13.5. Three Ctrl embryos and 4 Mut embryos were analyzed. **(b)** $CD68^{+}$ cells in the cardiac valves and cushion area were quantified from section immunofluorescence staining images. Three sections from each embryo were analyzed. There were no significant difference between Ctrl and Mut. Data are presented as mean values +/- SEM. *p*=0.1376, calculated from unpaired, two-tailed t-test. **(c)** Representative images of section immunohistochemical staining of mitral valves of control (Ctrl) and mutant (Mut) mice at P10. Five animals were analyzed per group. **(d)** $F4/80^{+}$ cells in the aortic and mitral valves were quantified from section immunohistochemical staining images. Cell numbers were counted per valve leaf. There were no significant difference between Ctrl and Mut. Data are presented as mean values +/- SEM. *p*=0.3027, calculated from unpaired, two-tailed t-test. Source data are provided as a Source Data file.

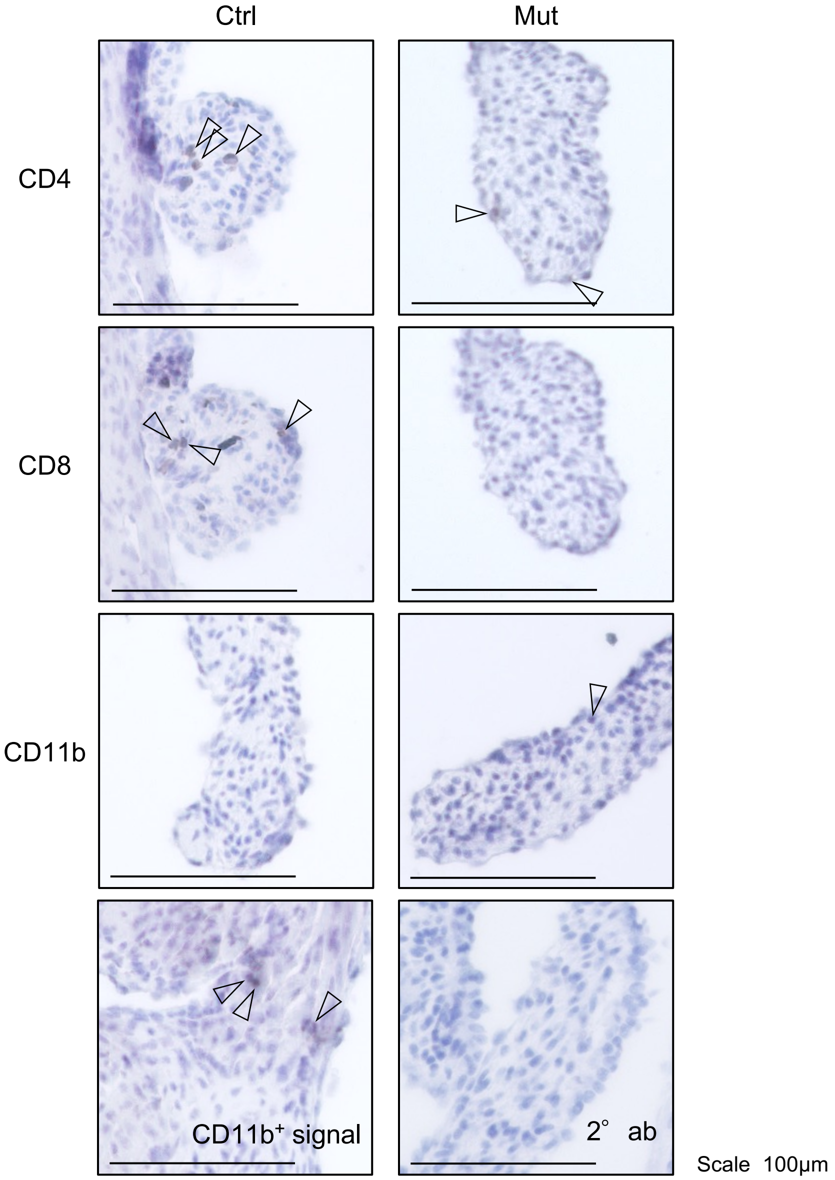

Supplementary Figure 10. Immunohistochemical staining of CD8, CD4, and CD11b to detect T cells and myeloid cells in P10 valves. The infiltration of these immune cells (indicated by arrow heads) was very minor in the valve region; CD11b-positive cells were rarely seen in the valves, but the presence of positive signals in other parts of the heart confirmed that the staining was successful. The negative control of the experiment without primary antibody (shown in the lower right corner panel) confirmed the absence of nonspecific signals. Four experiments were repeated with similar results.
