# Supplementary material for: Notch and retinoic acid signals regulate macrophage formation from endocardium downstream of Nkx2-5
Source: Nat Commun. 2023 Sep 5;14:5398. doi: 10.1038/s41467-023-41039-6 (PMC10480477; doi:10.1038/s41467-023-41039-6)
Supplement: Supplementary file 3 — Reporting Summary [file 41467_2023_41039_MOESM3_ESM.pdf]

nature portfolio

Corresponding author(s): *Double-anonymous peer review submissions: write DAPR and your manuscript number here instead of author names.*

Last updated by author(s): *YYYY-MM-DD*

Reporting Summary

Nature Portfolio wishes to improve the reproducibility of the work that we publish. This form provides structure for consistency and transparency in reporting. For further information on Nature Portfolio policies, see our Editorial Policies and the Editorial Policy Checklist.

Statistics

For all statistical analyses, confirm that the following items are present in the figure legend, table legend, main text, or Methods section.

n/a	Confirmed	
☐	☒	The exact sample size (*n*) for each experimental group/condition, given as a discrete number and unit of measurement
☐	☒	A statement on whether measurements were taken from distinct samples or whether the same sample was measured repeatedly
☐	☒	The statistical test(s) used AND whether they are one- or two-sided<br>*Only common tests should be described solely by name; describe more complex techniques in the Methods section.*
☒	☐	A description of all covariates tested
☐	☒	A description of any assumptions or corrections, such as tests of normality and adjustment for multiple comparisons
☐	☒	A full description of the statistical parameters including central tendency (e.g. means) or other basic estimates (e.g. regression coefficient) AND variation (e.g. standard deviation) or associated estimates of uncertainty (e.g. confidence intervals)
☐	☒	For null hypothesis testing, the test statistic (e.g. *F*, *t*, *r*) with confidence intervals, effect sizes, degrees of freedom and *P* value noted<br>*Give P values as exact values whenever suitable.*
☒	☐	For Bayesian analysis, information on the choice of priors and Markov chain Monte Carlo settings
☒	☐	For hierarchical and complex designs, identification of the appropriate level for tests and full reporting of outcomes
☒	☐	Estimates of effect sizes (e.g. Cohen's *d*, Pearson's *r*), indicating how they were calculated

Our web collection on statistics for biologists contains articles on many of the points above.

Software and code

Policy information about availability of computer code

Data collection	Section immunofluorescent staining were imaged using LSM880 confocal microscope (Zeiss)<br>Whole embryo or adult heart pictures were obtained using Discovery V12 SteREO microscope (Zeiss) and Stemi305 (Zeiss)<br>Immunohistochemistry and pentachrome staining image were obtained using BX51 (Olympus)<br>Hematopoietic colonies were counted under IX71 inverted system microscope (Olympus)<br>Flow cytometry was performed using MACSQuant (Miltenyi)<br>Pentachrome staining and immunohitochemical staining were imaged using BZ-9000 (Keyence)
Data analysis	The R package(version 4.1.2) Seurat (version 4.1) was used to analyze the scRNA-seq data<br>Metascape (URL https://metascape.org) version 3.5 was used for gene ontology analysis<br>Z-stack features were applied using ZEN 2.0 Black software (Zeiss)<br>Z projection of confocal imaging were performed using Imaris 8 software<br>Flow cytometry analysis was done using FlowJo (version 7)<br>Pentachrome staining and immunohistochemical staining images were analyzed using BZ-II Viwere/analyzer (Keyence)<br>Quantification of imaging data were performed using Fiji (ImageJ2, Version: 2.9.0)<br>Statistical analysis were performed on GraphPad Prism 7

For manuscripts utilizing custom algorithms or software that are central to the research but not yet described in published literature, software must be made available to editors and reviewers. We strongly encourage code deposition in a community repository (e.g. GitHub). See the Nature Portfolio guidelines for submitting code & software for further information.

Data

Policy information about availability of data

All manuscripts must include a data availability statement. This statement should provide the following information, where applicable:

- Accession codes, unique identifiers, or web links for publicly available datasets
- A description of any restrictions on data availability
- For clinical datasets or third party data, please ensure that the statement adheres to our policy

The scRNA-seq data that analyzed in this manuscript were obtained from the GEO database, accession number GSE76118, GSE126128, and GNomEx database under accession numbers 272R, 274R, 275-292R, 439R, and 440R.
The datasets generated during and/or analyzed during the current study are available from the corresponding author on reasonable request.

Research involving human participants, their data, or biological material

Policy information about studies with human participants or human data. See also policy information about sex, gender (identity/presentation), and sexual orientation and race, ethnicity and racism.

Reporting on sex and gender	*Use the terms sex (biological attribute) and gender (shaped by social and cultural circumstances) carefully in order to avoid confusing both terms. Indicate if findings apply to only one sex or gender; describe whether sex and gender were considered in study design; whether sex and/or gender was determined based on self-reporting or assigned and methods used. Provide in the source data disaggregated sex and gender data, where this information has been collected, and if consent has been obtained for sharing of individual-level data; provide overall numbers in this Reporting Summary. Please state if this information has not been collected. Report sex- and gender-based analyses where performed, justify reasons for lack of sex- and gender-based analysis.*
Reporting on race, ethnicity, or other socially relevant groupings	*Please specify the socially constructed or socially relevant categorization variable(s) used in your manuscript and explain why they were used. Please note that such variables should not be used as proxies for other socially constructed/relevant variables (for example, race or ethnicity should not be used as a proxy for socioeconomic status). Provide clear definitions of the relevant terms used, how they were provided (by the participants/respondents, the researchers, or third parties), and the method(s) used to classify people into the different categories (e.g. self-report, census or administrative data, social media data, etc.) Please provide details about how you controlled for confounding variables in your analyses.*
Population characteristics	*Describe the covariate-relevant population characteristics of the human research participants (e.g. age, genotypic information, past and current diagnosis and treatment categories). If you filled out the behavioural & social sciences study design questions and have nothing to add here, write "See above."*
Recruitment	*Describe how participants were recruited. Outline any potential self-selection bias or other biases that may be present and how these are likely to impact results.*
Ethics oversight	*Identify the organization(s) that approved the study protocol.*

Note that full information on the approval of the study protocol must also be provided in the manuscript.

Field-specific reporting

Please select the one below that is the best fit for your research. If you are not sure, read the appropriate sections before making your selection.

☒ Life sciences ☐ Behavioural & social sciences ☐ Ecological, evolutionary & environmental sciences

For a reference copy of the document with all sections, see nature.com/documents/nr-reporting-summary-flat.pdf

Life sciences study design

All studies must disclose on these points even when the disclosure is negative.

Sample size	Sample size for the embryonic analysis were empirically determined. Sample size for the colony assays, flow cytometry and immunostainings were determined by the power analysis with a type 1 error rate of 5%. Image figures are representative of the numbers indicated in the figure legend. Al cell and animal experiments were based on at least 3 biological replicates.
Data exclusions	No samples or animals were excluded.
Replication	Number of biological replicates were provided in the figure legends that contain quantitative analysis.
Randomization	Because the analyses were phenotypical analysis of embryos and their tissues or cells, randomization is not applicable.
Blinding	Investigators were blinded to allocation for outcome assessment.

Reporting for specific materials, systems and methods

We require information from authors about some types of materials, experimental systems and methods used in many studies. Here, indicate whether each material, system or method listed is relevant to your study. If you are not sure if a list item applies to your research, read the appropriate section before selecting a response.

Materials & experimental systems

n/a	Involved in the study
☐	☒ Antibodies
☐	☒ Eukaryotic cell lines
☒	☐ Palaeontology and archaeology
☐	☒ Animals and other organisms
☒	☐ Clinical data
☒	☐ Dual use research of concern
☒	☐ Plants

Methods

n/a	Involved in the study
☒	☐ ChIP-seq
☐	☒ Flow cytometry
☒	☐ MRI-based neuroimaging

Antibodies

Antibodies used

rat-anti-CD31 (1:200; BD Biosciences, Cat#553372, Clone: MEC13.3, Lot: 0307149), rat anti-CD41 (1:200; BD Biosciences, Cat#740504, Clone: MWReg30, Lot: 1105857), rabbit-anti-activated Notch1 (1:100; Abcam, Cat#ab52301), rat-anti-CD206 (1:200; Biolegend, Cat#141716, Clone: C068C2, Lot: B270129), rat anti-CD68 (1:200; Bio-Rad Laboratories, Cat#MCA1957GA, Lot: 158240), rabbit-anti-Dhrs3 (1:100; Proteintech, Cat#15393-I-AP, Lot: 00006856), rabbit-anti-cTnT (1:250, Sigma-Aldrich, Cat#SAB2108239, Lot: QC12352), rabbit-anti-RFP (1:200; Chromotek, Cat#5f8-100, Clone: 5F8, Lot: 90228002AB-15), mouse-anti-GFP (1:200; Novus Biologicals, Cat#NB600-597, Clone: 9F9F9, Lot: 31330), and mouse-anti-MF20 (1:500; deposited to the DSHB by Fischman, D.A. (DSHB Hybridoma Product MF 20)), CD45 (APC-Cy7-conjugated, 1:200; BD Biosciences, Cat#557659, Clone: 30-F11, Lot: 0300934), Cx3cr1 (APC-conjugated, 1:200; Biolegend, Cat#149008, Clone: SA011F11, Lot: B332498), CD4 (1:500,; Abcam, Cat#183685, Clone: EPR19514, Lot: 1000542-1), CD8 (1:500; Novus Biologicals, Cat#NBP1-49045SS, Clone: 53-6.7, Lot: FSGO62011A) and CD11b (PE-conjugated, 1:500; BD Biosciences, Cat#553311, Clone: M1/70, Lot: 1327971)
Alexa Fluor 488 goat anti- rat IgG(H+L) (1:1000, Invitrogen, Cat#A11006, Lot: 2416486), Alexa Fluor 488 goat anti- rabbit IgG(H+L) (1:1000, Life technologies, Cat#A11008, Lot: 1622775), Alexa Fluor 555 goat anti- rat IgG(H+L) (1:1000, Invitrogen, Cat#A21434, Lot: 2272647), Alexa Fluor 594 goat anti- rabbit IgG(H+L) (1:1000, Invitrogen, Cat#A11012, Lot: 2506100), Alexa Fluor 647 goat anti- rat IgG(H+L) (1:1000, Invitrogen, Cat#A21247, Lot: 2420724), Alexa Fluor 647 goat anti- rabbit IgG(H+L) (1:1000, Invitrogen, Cat#A21245, Lot: 2497486), Alexa Fluor 647 goat anti- mouse IgG(H+L) (1:1000, Invitrogen, Cat#A21235, Lot: 2284596)

Validation

Antibodies were validated by the respective vendors for the applications described in this manuscript. Antibodies used for Immunofluorescent staining were also referred from previously published data.

Eukaryotic cell lines

Policy information about cell lines and Sex and Gender in Research

Cell line source(s)

OP9 stromal cell line was a kind gift from Dr. Mikkola's laboratory at the University of California, Los Angeles (UCLA) (originally obtained from ATCC).

Authentication

Authentification was performed by supportive effect of OP9 on hematopoietic growth of the tissues explanted onto it.

Mycoplasma contamination

Mycoplasma levels were routinely tested in the cell line used and were mycoplasma free.

Commonly misidentified lines (See ICLAC register)

None

Animals and other research organisms

Policy information about studies involving animals; ARRIVE guidelines recommended for reporting animal research, and Sex and Gender in Research

Laboratory animals

All animal strains were maintained on a C57BL/6J background, with the exception of CAG-CAT-N1ICDtg mice, which was maintained on a mix background (ICR:C57BL/6J). Mice were genotyped using KOD FX Neo kit.
Mice information: Nkx2-5-cre (Moses et al, Genesis 2001), Rosa26-tdTomato (Jackson Laboratory, Strain#007908), Nkx2-5-flox (Pashmforoush et al, Cell, 2004), Rosa26-N1ICD-IRES-GFP (Murtaugh et al, PNAS 2003), CAG-CAT-N1ICDtg (Watanabe et al, Development, 2006), Csf1r-flox (Jackson Laboratory, Strain#021212)
Mouse housing conditions are as follows: Lights go on at 7am and off at 7pm. Humidity range is 45-55%. Temperature is maintained around 20-23°C.

Wild animals

No wild animals were used in this study

Reporting on sex	Males and females were used in approximately equal numbers for all experiments.
Field-collected samples	No field-collected samples were used in this study
Ethics oversight	All animal procedures conformed to the National Institutes of Health Guide for the Care and Use of Laboratory Animals and were approved by the Animal Research Committee at the Jikei University School of Medicine (approval no. 2020063) and UCLA Institutional Animal Care and Use Committee (protocol no. 2008-143).

Note that full information on the approval of the study protocol must also be provided in the manuscript.

Flow Cytometry

Plots

Confirm that:

- [x] The axis labels state the marker and fluorochrome used (e.g. CD4-FITC).
- [x] The axis scales are clearly visible. Include numbers along axes only for bottom left plot of group (a 'group' is an analysis of identical markers).
- [x] All plots are contour plots with outliers or pseudocolor plots.
- [x] A numerical value for number of cells or percentage (with statistics) is provided.

Methodology

Sample preparation	Hematopoietic progenitors in hearts of E10 embryos were detected with CD31 (FITC-conjugated, 1:200; BD Pharmingen) and CD41 (PE-conjugated, 1:200; Biolegend). Hematopoietic colonies generated from the heart, yolk sac, and caudal half of the embryo, including the AGM region, were collected from E8.5 embryos and washed with 5% FBS and PBS to make the single-cell suspension. Samples were blocked with mouse Fc block (BD Biosciences) for 5 min, followed by labeling for 25 min at 4°C with CD45 (APC-Cy7-conjugated, 1:200; BD Pharmingen) and Cx3cr1 (APC-conjugated, 1:200; Biolegend) antibodies for macrophage detection. Nonviable cells were excluded using 7-amino-actinomycin D (7-AAD, BD Biosciences). Cells were then analyzed by MACSQuant (Miltenyi Biotec, North Rhine-Westphalia, Germany) with the rat anti-monoclonal antibodies.
Instrument	MACSQuant (Miltenyi)
Software	FlowJo (version 7)
Cell population abundance	Flow cytometry was only used for analysis, no sorting.
Gating strategy	Cells were gated with forward and side scatter size selection, 7AAD detection of dead cells, and antibody staining.

- [x] Tick this box to confirm that a figure exemplifying the gating strategy is provided in the Supplementary Information.
